# Entamoeba histolytica HM-1: IMSS gene expression profiling identifies key hub genes, potential biomarkers, and pathways in Amoebiasis infection: a systematic network meta-analysis

**DOI:** 10.1042/BSR20220191

**Published:** 2022-10-21

**Authors:** Ram Nayan Verma, Md. Zubbair Malik, Naidu Subbarao, Gajendra Pratap Singh, Durgesh Nandini Sinha

**Affiliations:** 1 School of Computational and Integrative Sciences, Jawaharlal Nehru University, New Delhi 110067, India; 2 Department of Genetics and Bioinformatics, Dasman Diabetes Institute, Dasman, P.O. Box 1180, Kuwait city 15462, Kuwait; 3 Department of Mathematics, Temple University, Philadelphia, PA/Mercer County Community College, West Windsor, NJ, U.S.A.

**Keywords:** Amoebiasis infection, Differentially expressed genes (DEGs), Gene-interaction network

## Abstract

*Entamoeba histolytica* (*E. histolytica*) is an anaerobic parasite that causes Amoebiasis in the intestine or extraintestinal, with immunology, genetics, and environmental variables all playing a part in the disease’s development, but its molecular mechanism is unknown. One of the primary obstacles in understanding the etiology of Amoebiasis will be identifying the genetics profiling that controls the Amoebiasis network. By examining the gene expression profile of Amoebiasis and comparing it with healthy controls, we could identify differentially expressed genes (DEGs). DEGs were used to build the Amoebiasis protein interaction network and calculated its network topological properties. We discovered nine key hub genes (KHGs): JUN, PTGS2, FCGR3A, MNDA, CYBB, EGR1, CCL2, TLR8, and LRRK2 genes. The genes JUN and EGR1 were transcriptional factors (TFs) and up-regulated, others down-regulated. hsa-miR-155-5p, hsa-miR-101-3p, hsa-miR-124-3p, hsa-miR-26b-5p, and hsa-miR-16-5p are also among the essential miRNAs that have been demonstrated to be targeted by KHGs. These KHGs were primarily enriched in the IL-17 signaling pathway, TNF signaling pathway, NOD-like receptor signaling pathway, and Toll-like receptor signaling pathway. miRNAs were grouped in various pathways, focusing on the TGF-β signaling pathway, human immunodeficiency virus 1 infection, insulin signaling pathway, signaling pathways regulating pluripotency of stem cells, etc. Amoebiasis KHGs (JUN, PTGS2, CCL2, and MNDA) and their associated miRNAs are the primary targets for therapeutic methods and possible biomarkers. Furthermore, we identified drugs for genes JUN, PTGS2, FCGR3A, CCL2, and LRRK2. KHGs, on the other hand, required experimental validation to prove their efficacy.

## Introduction

*Entamoeba histolytica* (*E. histolytica*) is an anaerobic parasite that causes Amoebiasis in the intestine or extraintestinal [1,2]. Amoebiasis is projected to affect 50 million people worldwide yearly, resulting in 55,000 fatalities [3]. *Entamoeba* *histolytica* resides mainly in the large intestine and causes no symptoms. It has a biphasic life cycle, with a latent cyst stage resistant to the environment and transmits the infection. The other type of proliferative trophozoite is motile and produces illness [4]. Infectious cysts spread by the feces-to-mouth pathway, excysting in the terminal ileum and releasing invasive trophozoites [5].

The pathogenicity of this anaerobic parasite depends on its ability to cling to the intestinal epithelium and degrade extracellular matrix proteins, causing tissue lesions that lead to abscesses and a host acute inflammatory response [6–8]. The mechanisms of pathogenicity, virulence factors, metabolism, and the mounted host immunological response have all been widely explored. However, complex host–parasite interaction during distinct stages of the disease remains a significant bottleneck for developing effective therapeutics and diagnostics [9].

To kill the host intestinal mucosa and induce sickness, *E. histolytica* uses a variety of ways [10]. The attachment of trophozoites to host mucous and epithelial cells in the colon is thought to be mediated by multisubunit amebic GaINAc lectin after excystation within the small intestinal lumen. Trophozoites also secrete a number of cysteine proteases that break down mucin and the extracellular matrix [11,12]. Contact-dependent death of resident host cells is still a mystery. Host cells appear to break the cell membrane due to fast fluctuations in intracellular calcium levels, which leads to host cell alterations that resemble apoptosis, such as membrane blebbing, DNA digestion, and caspase activation [13]. Finally, *E. histolytica* trophozoites phagocytize the host’s nucleated and red blood cells. Virulence is significantly associated with the ability to phagocytize host cells [14].

Amoebiasis is being treated with metronidazole as a first-line medication. Metronidazole resistance has been linked to higher levels of iron-containing superoxide dismutase and peroxiredoxin and lower levels of ferredoxin and flavin reductase [15]. The reduced expression of drug targets such as methionine γ-lyase has been associated with trifluoro methionine resistance [16,17].

Human resistance or susceptibility to *E. histolytica* infection has been studied, but the results are contradictory. The application of whole blood transcriptional profiling in humans has recently led to a better knowledge of the host response to infectious disease and the identification of blood signatures and possible biomarkers for diagnosis, prognosis, and treatment monitoring. The study of the host transcriptome has been widely used to investigate the intricate interactions between humans and microorganisms [18,19].

This technique successfully discovered neutrophil-driven interferon (INF)-inducible blood transcriptional signature for active Tuberculosis that involved both INF- and type I IFN-α/β signaling, which was later verified in multiple countries [20]. This neutrophil-driven interferon signature was found in active disease but not a latent infection or healthy controls. While patients with the autoimmune disease systemic lupus erythematosus had an IFN-inducible signature, variances in signatures separated the two profiles. INF-inducible gene expression is extensively defined by viral and bacterial infections such as melioidosis. Whole blood signatures distinguish between bacterial and viral illnesses and different viral infections [21]. Blood transcriptional patterns that can discriminate between rapid and slow disease progression have been established in HIV [22]. Other signs identify pulmonary from extrapulmonary tuberculosis and pulmonary tuberculosis from pulmonary sarcoidosis, pneumonia, and lung tumors. As a result, transcriptional fingerprints that can track treatment effectiveness are an essential weapon in the fight against infectious illnesses. A study from two South African cohorts studied longitudinally shows that the transcriptional signature of active tuberculosis disease quickly reduces with successful therapy [23].

The human host response to protozoan diseases such as plasmodium species-caused malaria [24,25] and Trypanosoma cruzi-causes Chagas disease has been studied using whole blood transcriptional profiling [26]. In animal models and human investigations of leishmaniases, expression profiling has also been used. Whole blood transcriptomics was utilized to examine expression profiles in patients with active visceral leishmaniasis caused by *Leishmania infantum*, asymptomatic infected individuals, VL patients in remission, and controls [27].

We investigated differentially expressed host genes better to understand the immunologic aspects of *E. histolytica* infection and discover possible treatment targets that could distinguish diseased from non-diseased patients. The relevance of taking a systematic approach to dealing with this underlying complexity can be seen in the interconnected and intrinsically complex biochemical network of disease states. A comprehensive analysis of insight derived from various levels in the omics chain can cumulatively elucidate the variations that are efficiently interpreted from the genome and how it influences its concomitant at the molecular level, thus providing an extensive and integrated perspective on the stacked changes that occur upon infection, as well as on the ensuing therapeutic intercession.

The disease’s etiology is imprecise due to a lack of an excellent quick diagnosis system and our reliance on the existing standard single-gene screening pattern. Human antibodies have a hard time entering the cell since *E. histolytica* is an intracellular infection. Nonetheless, the cell-mediated immune response plays a critical role in pathogen containment and the progression of infection to active illness. Construction of a gene interaction network of differentially expressed genes (DEGs) involved in the immune response to Amoebiasis and screening to look for therapeutic targets (gene expression biomarkers) can play a significant role in determining the fate of infection by developing a method of discovering new effective drugs and the earliest diagnosis in susceptibility to Amoebiasis disease to reduce the burden of suffering, among other things.

This study performed a meta-analysis by comparing gene expression profiling of active *E. histolytica* infected data with data from healthy individuals and identified disease-specific DEGs, stipulating a salient molecular network and revealing the host consequences of infection in this population. To further our research, we integrated curated and empirically validated connections in humans to develop protein–protein (or, more specifically, gene–gene) interaction networks. The centralities of the networks in this network were also measured, and the genes in the network were ranked according to their centralities [28]. A gene-transcription factors (TFs) regulation network comprising key hub genes (KHGs) was also evaluated to identify the relationships between TFs and KHGs. The findings of this study are intended to improve our understanding of the genes or proteins involved in the establishment and development of Amoebiasis, allowing for more effective treatment options. This investigation will reveal novel insights into the host immunological systems involved in *E. histolytica* infection and new genes that may be used to distinguish active Amoebiasis patients from those who do not have the illness.

## Materials and methods

**Figure 1:**
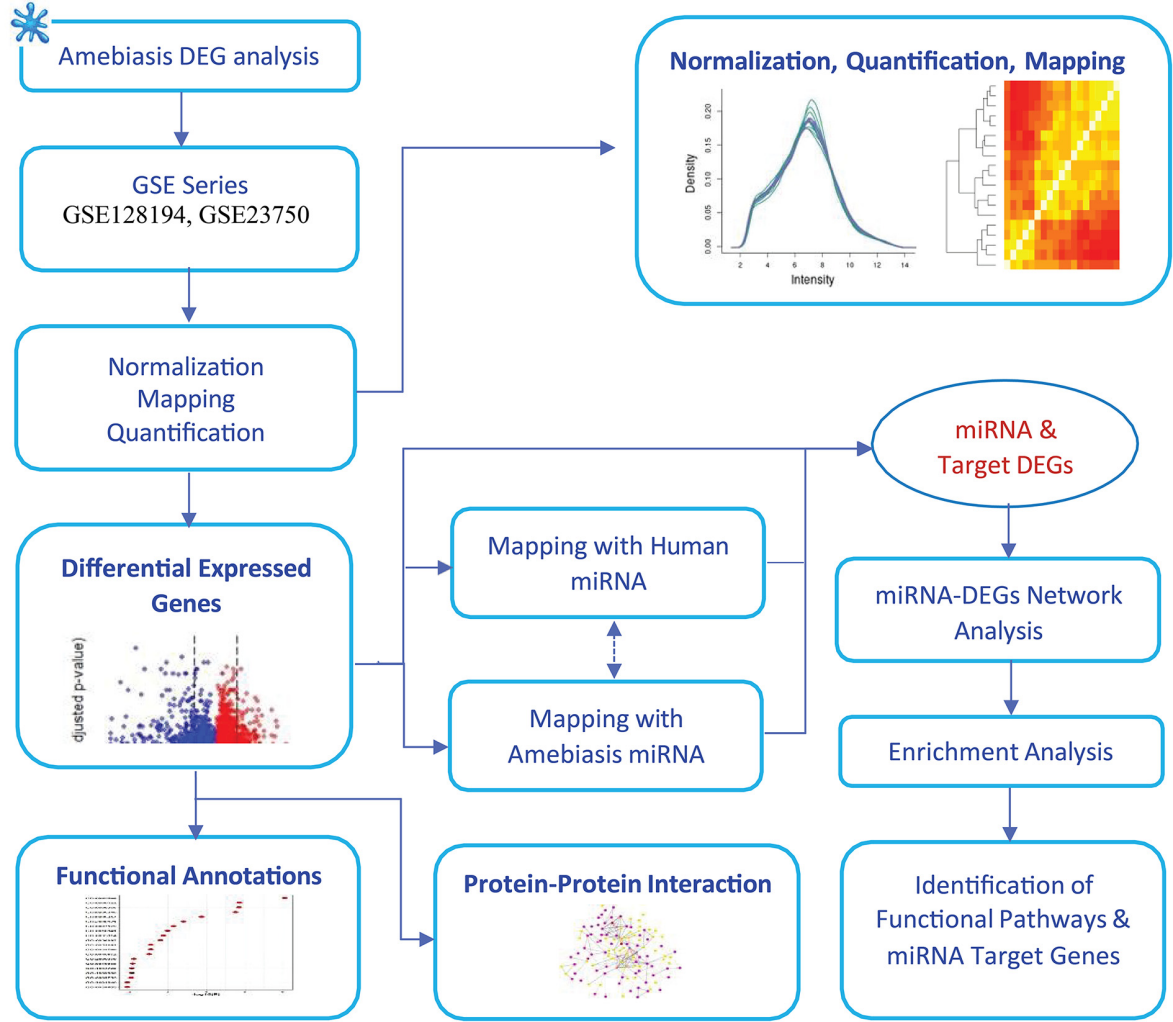
Schematic diagram of the workflow Detailed workflow of methods implemented in Amoebiasis infection-associated PPI network study.

The detailed workflow is as shown in Figure 1.

### Preprocessing and acquisition of datasets

Two separate RNA-Seq datasets, GSE128194 and GSE23750, were downloaded from the NCBI GEO repository database (https://www.ncbi.nlm.nih.gov/geo/) to evaluate the human gene expression profiling in active (*E. histolytica* infected) and healthy cases. Table 1 shows the datasets in detail. GEO datasets of active Amoebiasis and healthy controls were used to recruit participants for this investigation. We didn’t include any samples treated with a drug or were linked to another disease. R was used to preprocess the sample data, normalize it, and remove non-expressed probes/genes.

**Table 1:** GSE datasets used in the present study

S.No.	GSE Series ID	Samples	Platforms	Methods	References
1	GSE128194	03HC,03EH	GPL16791	Illumina	[29]
2	GSE23750	08HC,08EH	GPL6244	Affymetrix	[30]

EH, Amoebiasis (*E. histolytica*); HC, healthy control.

### Data analysis and visualization of DEGs

RStudio (version 1.3.1093) [31] and R Version 3.4.4 [32] were used to perform all data analysis. To avoid inconsistencies, the Bioconductor software Lumi [33] was used to read raw Illumina GSE series expression data and perform background data correction and quantile standardization. For background data correction and normalization of Affymetrix GSE series samples, we used the robust multi-array average (RMA) [34] technique using the affy-package [35] of R. In this investigation. We used the LIMMA [36] package to analyze data and quantify DEGs by measuring gene expression values (DEGs). It estimates f-test and t-test (simple and moderate) using linear modeling (LM) and empirical Bayes (EM) methods and lessens the standard errors to provide stable and reproducible results [37]. DEGs analysis was performed for comparison between control and clinical groups. R package *ggplot2(3.1.2)* [38] was used for data visualization. The threshold values for differential expression were considered at least a log_2_(fold change, *FC*) compared with healthy controls. The threshold for determining the fold change for each gene was set to Benjamini–Hochberg adjusted *P*-value (*P*_adj_) ≤ 0.05 and *P*-value ≤ 0.05.

### Probe IDs conversion, network construction, and visualization

The probe IDs of the gene expression profiles were also converted to their appropriate gene symbols using the g: Profiler online server (http://biit.cs.ut.ee/gprofiler/convert) [39]. We also used the STRING v11 (Search Tool for the Retrieval of Interacting Genes/Proteins) database (https://string-db.org/) [40] to build a protein–protein interaction (PPI) network of DEGs (up- and down-regulated) in humans, retaining curated and experimentally validated interactions. In Cytoscape-3.7.1, a network was displayed [41].

### Functional analysis/GO enrichment

To produce a list of biologically significant DEGs, the DAVID 6.8 Web server (https://david.ncifcrf.gov/) [42] is used. It is an open bioinformatics resource that can be accessed via a web browser and provides investigators with a comprehensive collection of functional annotation tools to help them comprehend the biological meaning of a considerable number of genes. DAVID tools can (1) identify enriched biological themes, such as gene ontology (GO) terms, (2) discover enriched functional-related gene groups, (3) visualize genes on BioCarta and KEGG pathway maps, and (4) display related many-genes-to-many-terms in a 2D view and search for other functionally related genes, not in the list for any given gene list.

### Identify key hub genes

A systematic analysis is required to gain maximal insights from a biological network that has been constructed as described. Identifying the KHGs (molecular regulators) is a primary goal in omics data analysis. Finding KHGs in DEGs network (GIN) using topological network properties defines different measures of a node’s connectivity. These topological properties include degree and centrality measures such as closeness centrality, eigenvector centrality, and betweenness centrality. Nodes having high betweenness centrality known as bottlenecks have been shown to be predictive of gene essentiality. These statistical properties were estimated using the *Network Analyzer* plug-in Cytoscape-3.7.1.

TFs are critical trans-acting factors in transcriptional regulation. Understanding the regulation mechanism in human illness requires elucidating the TF-target interaction. TRRUST (v2.0) server (https://www.grnpedia.org/trrust/) [43], a manually curated database of human transcriptional regulatory networks containing 8444 TF-target regulatory interactions of 800 individuals, was utilized to evaluate all of our KHGs’ major TFs.

## Results

### Analysis and results

**Figure 2:**
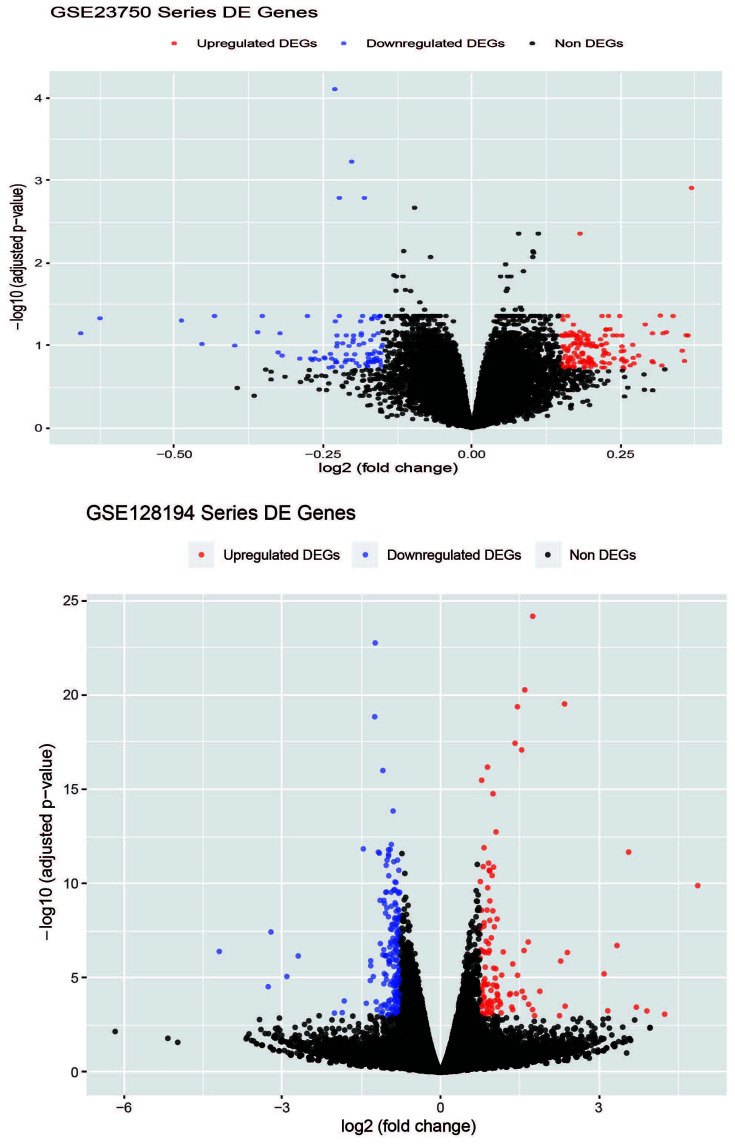
Volcano plot of the down-regulated and up-regulated DEGs The volcano plot shows the down-regulated and up-regulated DEGs of Amoebiasis infected and healthy cases. The *x*-axis represents the log_2_(*FC*) and the *y*-axis represents −log_10_ (*P-*values).

Differential expression analysis (volcano plot for all samples are shown in (Figure 2) revealed 314 DEGs, with 196 down-regulated and 118 up-regulated in a high throughput sequencing of Amoebiasis using a 3D model of the human intestine series *GSE128194* of expression profiling by an array with the threshold cut-offs log_2_ |*FC*| > 1.5, *P*-value* <* 0.05 and *P*_adj_ ≤ 0.05. The *GSE23750* series expression profiling by the array for differential expression was used to access gene differences after *E. histolytica* infection. The threshold cut-off *P-*value < 0.001, *P*_adj_ ≤ 0.05 with log_2_ |*FC*| ≥ 1 uncover 56 up-regulated and 92 down-regulated genes.

**Figure 3:**
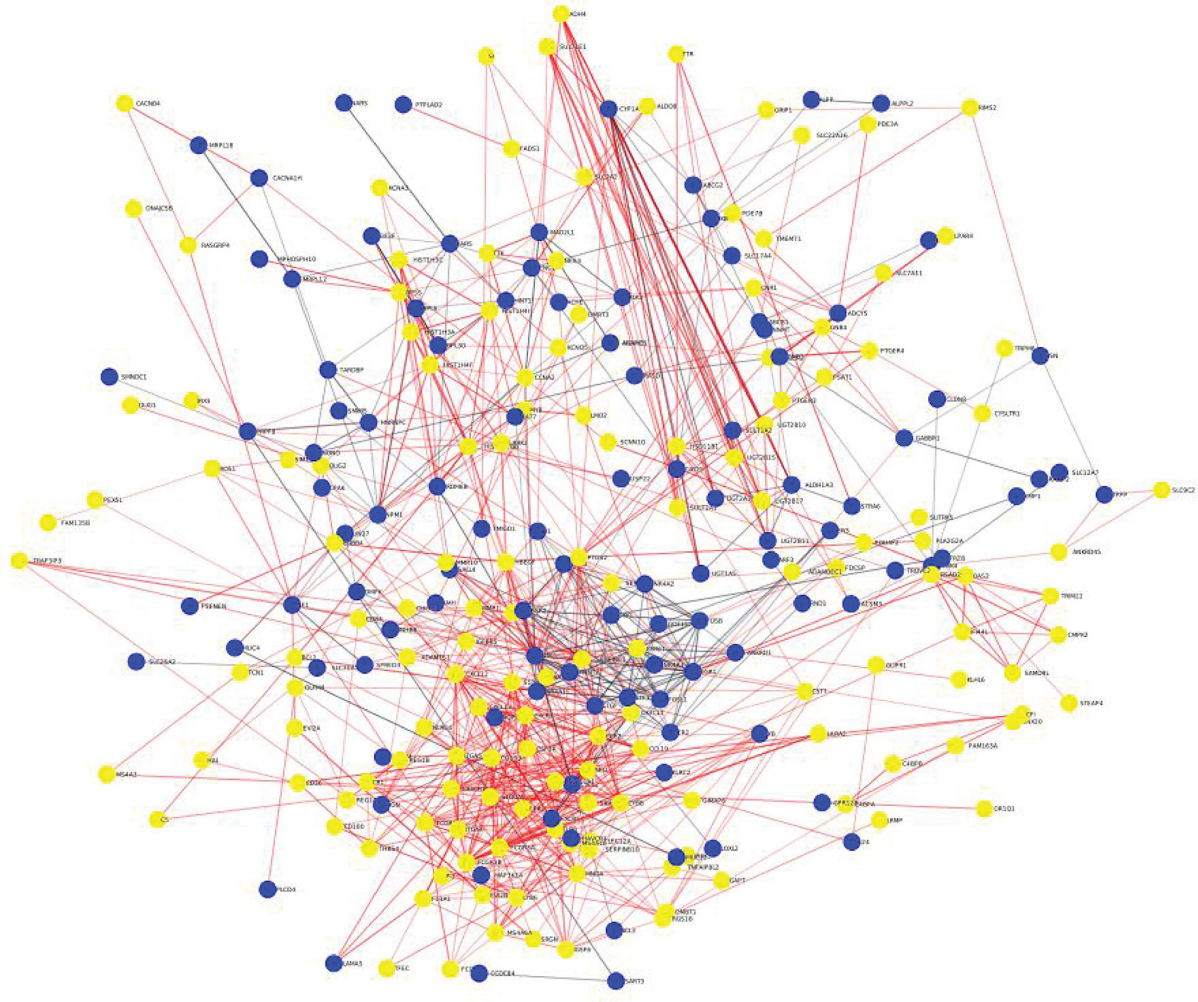
PPI network of DEG Up- and down-regulated genes are represented as yellow and blue colors, respectively.

Further, a total of 193 up-regulated and 262 down-regulated genes with cut-off threshold values log_2_ |*FC*| ≥ 1, *P-*value ≤ 0.05, *P*_adj_ ≤ 0.5 were identified in both datasets. Moreover, using the server (http://biit.cs.ut.ee/gprofiler/convert), g: Profiler online server, all probe IDs of the genes were transformed into their corresponding gene symbols. And unique gene symbols were identified among all sets of expressed genes, as listed in Supplementary files (S1, S2, S3, S4). Next, PPI networks were constructed from a *string-db* database of DEGs (Figure 3).

#### Annotation of DEGs’ functions

To better understand the biological activities of DEGs, we used a GO enrichment analysis. The KEGG pathway and Figures 5 and 6 depict the considerable enrichment of down-regulated and up-regulated DEGs.

**Figure 4:**
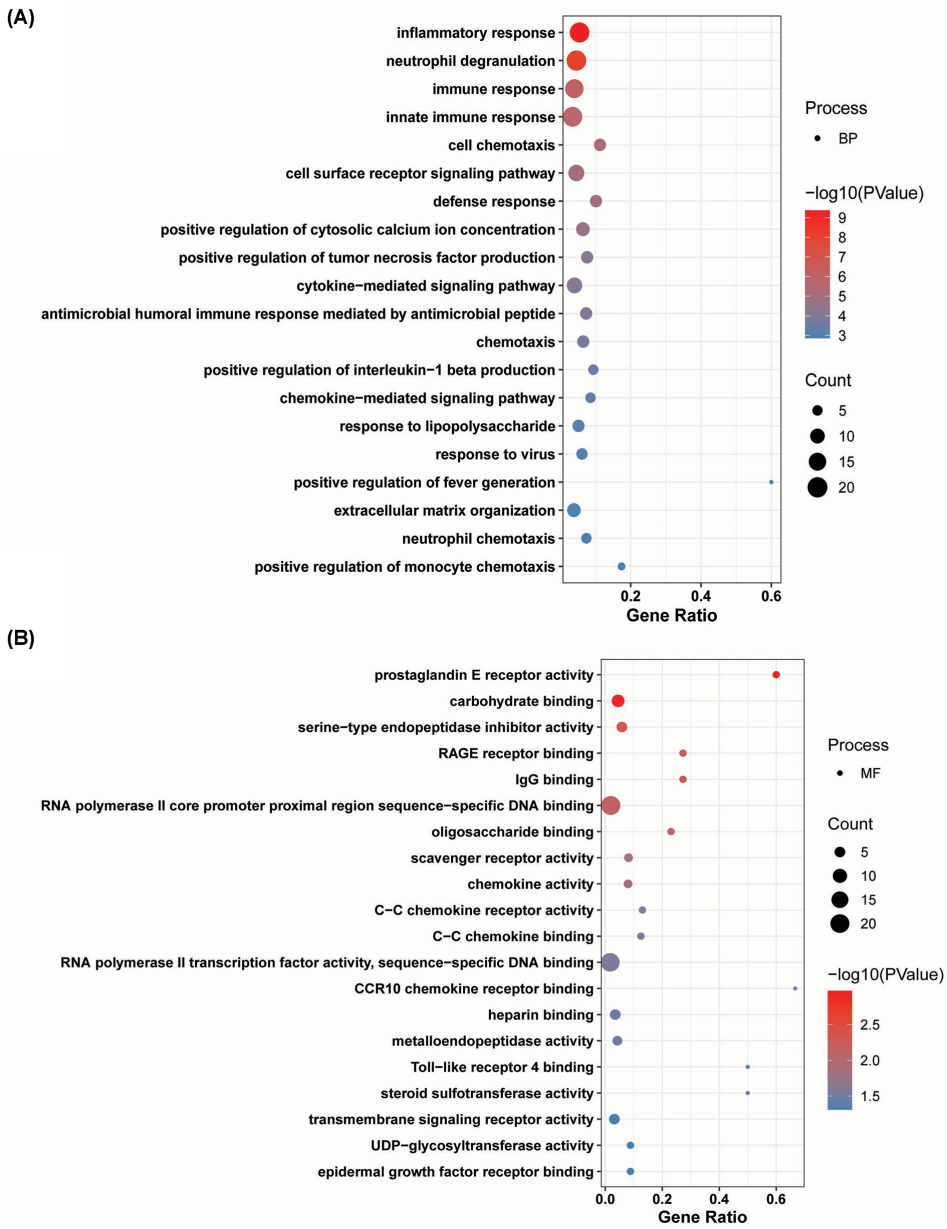
GO analysis of down-regulated DEGs The GO of DEGs. The *x*-axis represents the gene ratio. The color of the dots represents the −log_10_(*P*) and the size of the dots represents the gene count. (**A**) Biological process and (**B**) molecular function.

Biological processes were overrepresented among the commonly down-regulated DEGs (BP), including inflammatory response, neutrophil degranulation, immune response, innate immune response, cell chemotaxis, cell surface receptor signaling pathway, defense response, positive regulation of cytosolic calcium ion concentration, positive regulation of tumor necrosis factor production, cytokine-mediated signaling pathway, antimicrobial humoral immune response mediated by antimicrobial peptide, chemotaxis, positive regulation of interleukin-1β production, chemokine-mediated signaling pathway, response to lipopolysaccharide, response to the virus, positive regulation of fever generation, extracellular matrix organization, neutrophil chemotaxis, and positive regulation of monocyte chemotaxis (Figure 4A).

**Figure 5:**
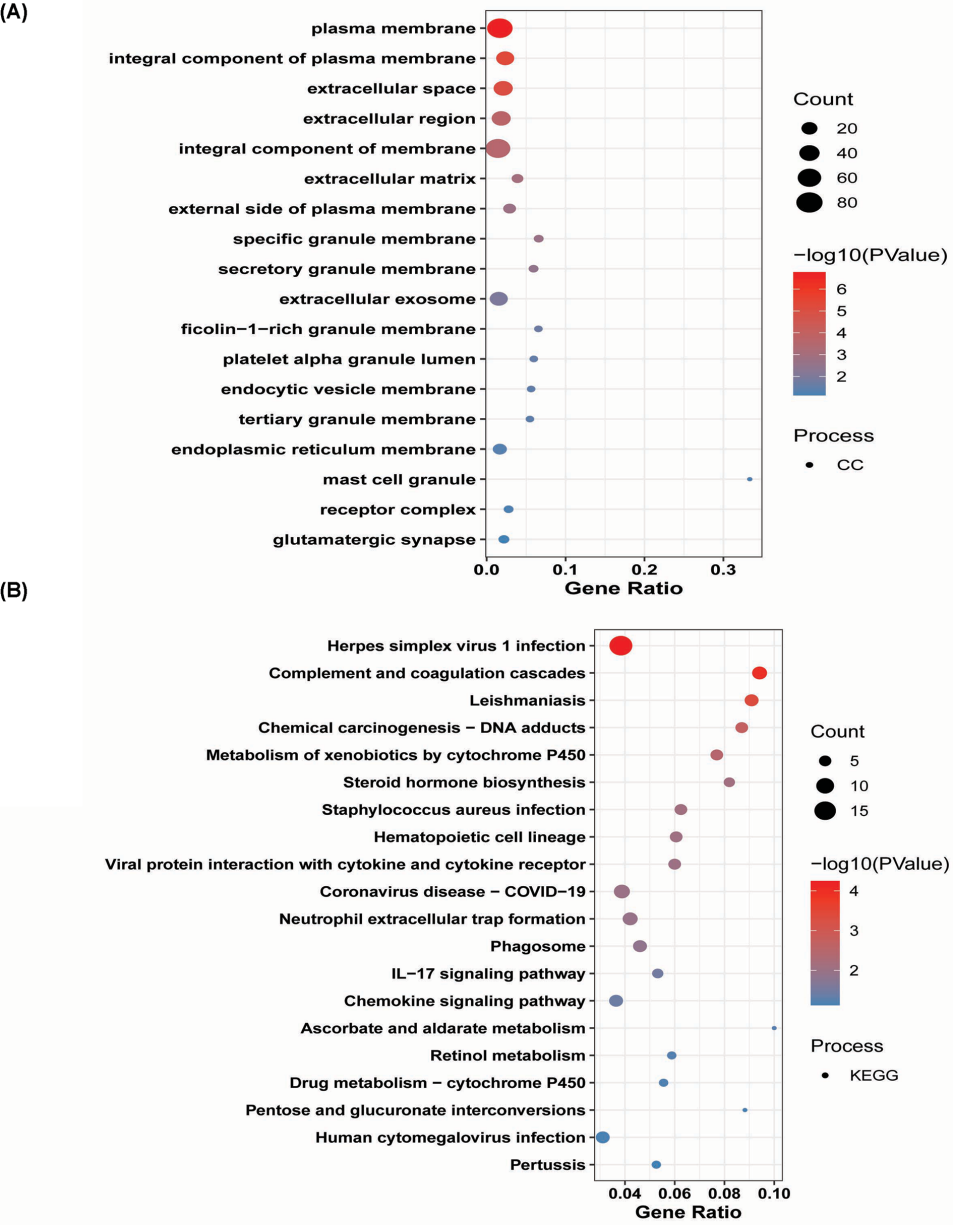
GO analysis of down-regulated DEGs (**A**) Cellular component and (**B**) KEGG pathways

**Figure 6:**
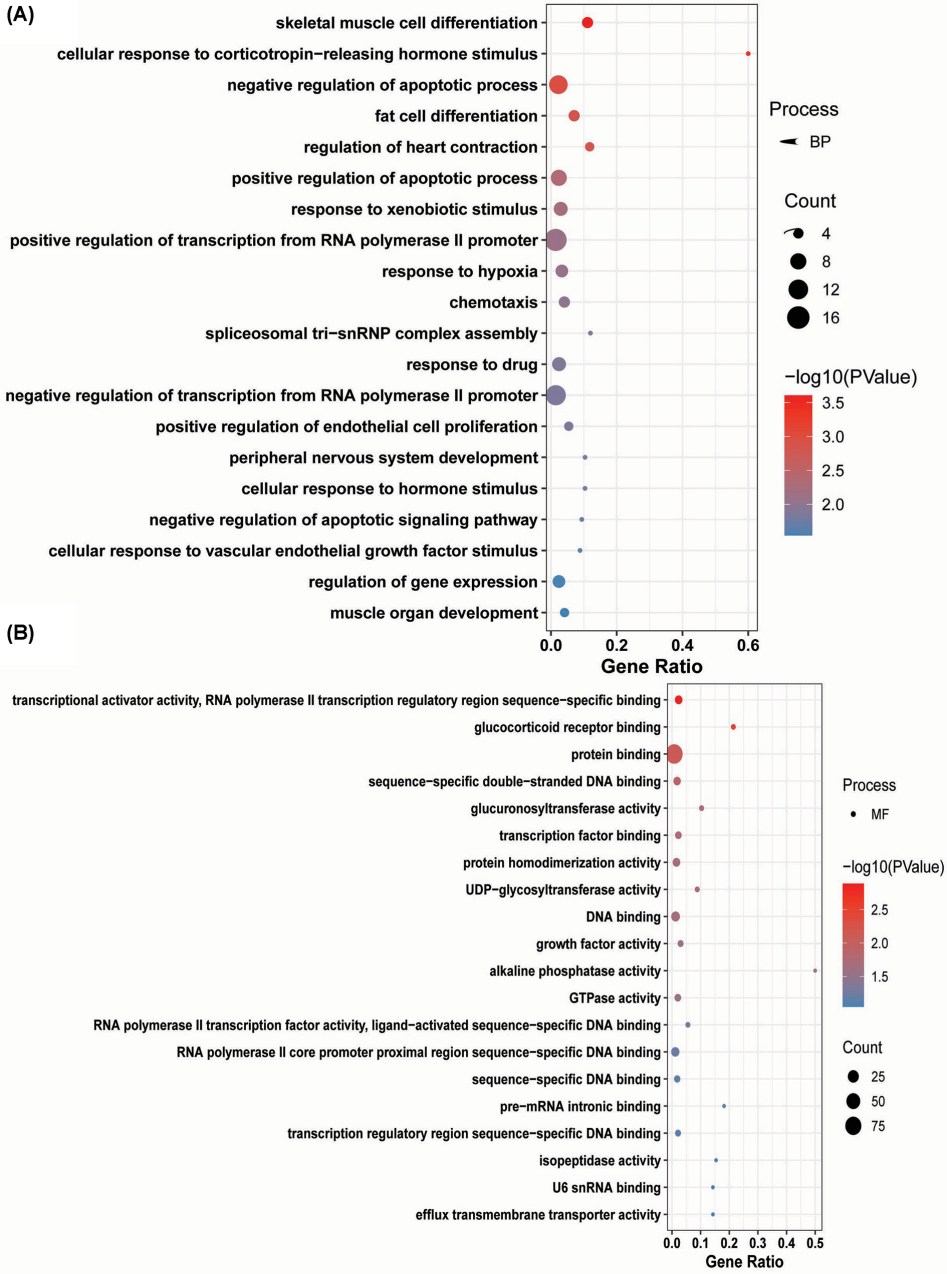
GO analysis of up-regulated DEG sets The *x*-axis represents the gene ratio. The color of the dots represents the −log_10_(*P-*value), and the size of the dots represents the gene count. (**A**) Biological process and (**B**) molecular function.

The MF of the down-regulated gene was primarily enriched in prostaglandin E receptor activity, carbohydrate-binding, serine-type endopeptidase inhibitor activity, IgG binding, RAGE receptor binding, RNA polymerase II core promoter proximal region sequence-specific DNA binding, oligosaccharide binding, scavenger receptor activity, chemokine activity, C-C chemokine receptor activity, C-C chemokine binding, RNA polymerase II TF activity, sequence-specific DNA binding, CCR10 chemokine receptor binding, heparin-binding, metalloendopeptidase activity, steroid sulfotransferase activity, Toll-like receptor 4 binding, transmembrane signaling receptor activity, epidermal growth factor receptor binding, and UDP-glycosyltransferase activity (Figure 4B). The changes in cellular components (CC) of DEGs that were down-regulated mainly enriched in plasma membrane, integral component of plasma membrane, extracellular space, extracellular region, integral component of membrane, extracellular matrix, external side of plasma membrane, specific granule membrane, secretory granule membrane, extracellular exosome, ficolin-1-rich granule membrane, plateletα granule lumen, endocytic vesicle membrane, tertiary granule membrane, endoplasmic reticulum membrane, mast cell granule, receptor complex, glutamatergic synapse (Figure 5A).

The down-regulated DEGs were mainly enriched, according to biological pathway analysis in Herpes simplex virus 1 infection, complement and coagulation cascades, Leishmaniasis, chemical carcinogenesis – DNA adducts, metabolism of xenobiotics by cytochrome P450, steroid hormone biosynthesis, *Staphylococcus aureus* infection, hematopoietic cell lineage, viral protein interaction with cytokine and cytokine receptor, Coronavirus disease – COVID-19, neutrophil extracellular trap formation, phagosome, IL-17 signaling pathway, chemokine signaling pathway, ascorbate and aldarate metabolism, retinol metabolism, drug metabolism – cytochrome P450, pentose and glucuronate interconversions, human cytomegalovirus infection, and pertussis (Figure 5B).

Up-regulated DEGs were enriched in BP, including skeletal muscle cell differentiation, cellular response to corticotropin-releasing hormone stimulus, negative regulation of apoptotic process, fat cell differentiation, regulation of heart contraction, positive regulation of apoptotic process, response to xenobiotic stimulus, positive regulation of transcription from RNA polymerase II promoter, response to hypoxia, chemotaxis, spliceosomal tri-snRNP complex assembly, response to drug, negative regulation of transcription from RNA polymerase II promoter, positive regulation of endothelial cell proliferation, cellular response to hormone stimulus, peripheral nervous system development, negative regulation of apoptotic signaling pathway, cellular response to vascular endothelial growth factor stimulus, regulation of gene expression, and muscle organ development (Figure 6A). The molecular function of up-regulated DEGs was transcriptional activator activity, RNA polymerase II transcription regulatory region sequence-specific binding, glucocorticoid receptor binding, protein binding, sequence-specific double-stranded DNA binding, glucuronosyltransferase activity, TF binding, protein homodimerization activity, UDP-glycosyltransferase activity, DNA binding, growth factor activity, alkaline phosphatase activity, GTPase activity, RNA polymerase II TF activity, ligand-activated sequence-specific DNA binding, RNA polymerase II core promoter proximal region sequence-specific DNA binding, sequence-specific DNA binding, pre-mRNA intronic binding, transcription regulatory region sequence-specific DNA binding, isopeptidase activity, efflux transmembrane transporter activity, U6 snRNA binding, etc. (Figure 6B).

**Figure 7:**
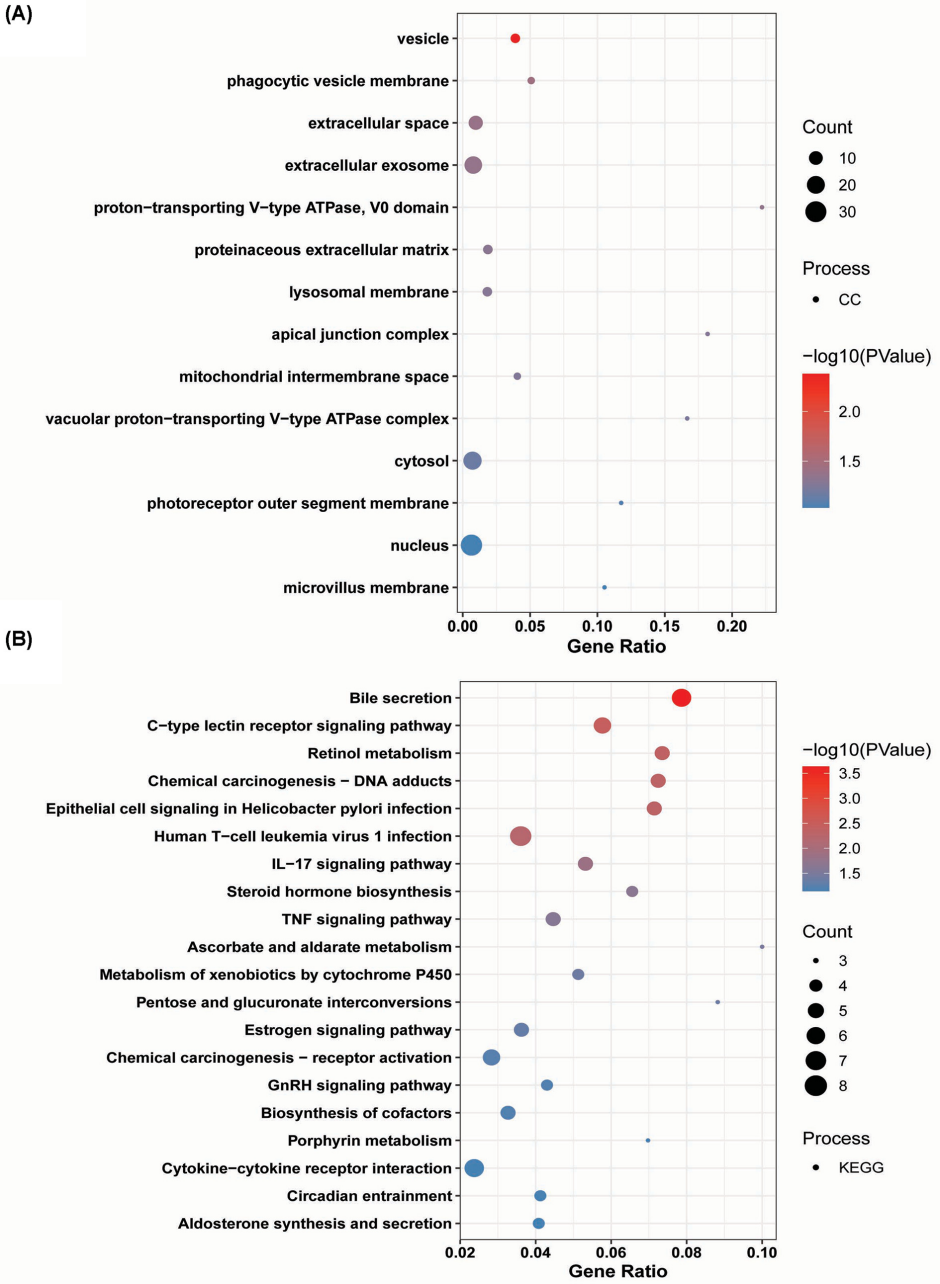
GO analysis of up-regulated DEG sets The *x*-axis represents the gene ratio. The color of the dots represents the −log_10_(*P*-value) and the size of the dots represents the gene count. (**A**) Cellular component and (**B**) KEGG pathways.

Changes in cellular components of up-regulated DEGs were mainly vesicle phagocytic vesicle membrane, extracellular space, extracellular exosome, proton-transporting V-type ATPase, V0 domain, proteinaceous extracellular matrix, lysosomal membrane, apical junction complex, mitochondrial intermembrane space, vacuolar proton-transporting V-type ATPase complex, cytosol, photoreceptor outer segment membrane, nucleus, microvillus membrane, etc. (Figure 7A).

Biological pathway analysis revealed the up-regulated DEGs were mainly enriched in Bile secretion, C-type lectin receptor signaling pathway, retinol metabolism, chemical carcinogenesis-DNA adducts, epithelial cell signaling in *Helicobacter pylori* infection, human T-cell leukemia virus 1 infection, IL-17 signaling pathway, steroid hormone biosynthesis, TNF signaling pathway, ascorbate and aldarate metabolism, metabolism of xenobiotics by cytochrome P450, pentose and glucuronate interconversions, estrogen signaling pathway, chemical carcinogenesis-receptor activation, GnRH signaling pathway, biosynthesis of cofactors, porphyrin metabolism, cytokine–cytokine receptor interaction, circadian entrainment, aldosterone synthesis and secretion, etc (Figure 7B).

#### Identifying important key hub genes through network analysis

To choose the most central nodes inside the network, centrality analyses each node based on degree, betweenness, closeness, and eigenvector. It was used to identify KHGs and bottleneck genes in scale-free biological networks based on network topological properties. A node with a more excellent centrality value can assist in identifying a biological entity that plays the most significant part in the activities of the biological network. So, to enlist inferred genes in this network, we chose the top 20 ranking genes based on degree, betweenness, closeness, and eigenvector centralities as listed in Table 2, and applying CytoHubba, we enlisted top 20 ranking genes based on MCC, DMNC MNC, and EPC properties as listed in Table 3, then looked for common genes in at least three properties among the top 20 rankings across all evaluated centralities, which we regarded as KHGs. JUN, PTGS2, FCGR3A, MNDA, CYBB, EGR1, CCL2, TLR8, and JLRRK2 were frequent and assumed KHGs.

**Table 2:** Genes inferred from DEG sets’ gene interaction networks

S.No.	Degree [*P*(*k*)]	Betweenness centrality [*C*_B_ (*k*)]	Closeness centrality [*C*_C_ (*k*)]	Eigenvector centrality [*C*_E_ (*k*)]
**1**	JUN	PTGS2	JUN	PTGS2
**2**	PTGS2	JUN	PTGS2	JUN
**3**	FCGR3A	LRRK2	CYBB	LRRK4
**4**	FCGR3B	CYBB	CXCY12	CCL2
**5**	CXCL12	CYP1A1	LRRK2	MYB
**6**	SELL	EGR1	CTGF	CYP1A1
**7**	CYBB	S100A9	FCGR3A	EGR1
**8**	MNDA	ENO2	CCL2	CXCR2
**9**	EGR1	NF1	SERPINE1	NF1
**10**	CCL2	CNR1	EGR1	SIGLEC1
**11**	TLR8	TLR8	HBEGF	ADH4
**12**	CD163	MYB	CD163	CX3CL1
**13**	CTGF	GNB3	TLR8	ENO2
**14**	SERPINE1	CCNA2	CXCL2	S100A9
**15**	CXCL2	CSF3R	FCGR3B	CNR1
**16**	SIGLEC1	GIG2J	CX3CL1	PTGER2
**17**	LRRK2	CR1	S100A8	GST7
**18**	CX3CR1	RPS5	SELL	MNDA
**19**	CX2CL1	MNDA	MMP1	GIG25
**20**	FCGR1A	FCGR3A	NMDA	FCGR3A

And inferred as KHGs.

**Table 3:** Genes inferred from DEG sets’ gene interaction networks based on MCC, DMNC, MNC, and EPC

S.No.	MCC	DMNC	MNC	EPC
**1**	JUN	NR4A3	JUN	FCGR3A
**2**	EGR1	EGR3	FCGR3A	CXCL12
**3**	ATF3	NR4A1	FCGR3B	CCL2
**4**	FOSB	S100A9	CXCL12	PTGS2
**5**	NR4A1	FOSB	PTGS2	JUN
**6**	EGR2	CCL19	CYBB	SELL
**7**	EGR3	NR4A2	SELL	CYBB
**8**	FCGR3A	CX3CL1	MNDA	FCGR3B
**9**	FCGR3B	HIST1H3C	CCL2	TLR8
**10**	NR2A2	FCGR1B	TLR8	CD163
**11**	NR4A3	IER2	CD163	MNDA
**12**	TLR8	EGR2	EGR1	CX3CR1
**13**	CYBB	FCGR1A	CTGF	CXCL2
**14**	FCGR1A	MMP10	SERPINE1	CX3CL1
**15**	CCL2	SAMD9L	CXCL2	SIGLEC1
**16**	SIGLEC1	FPR1	FCGR1A	EGR1
**17**	CD163	SERPINB2	CX3CR1	FCGR1A
**18**	SELL	TR1M22	MMP1	HBEGF
**19**	S100A9	CMPK2	CX3CL1	CTGF
**20**	CXCL12	ATF3	FPR1	SERPINE1

Abbreviations: DMNC, density of maximum neighborhood component; EPC, edge percolated component; MCC, maximal clique centrality; MNC, maximum neighborhood component.

**Figure 8:**
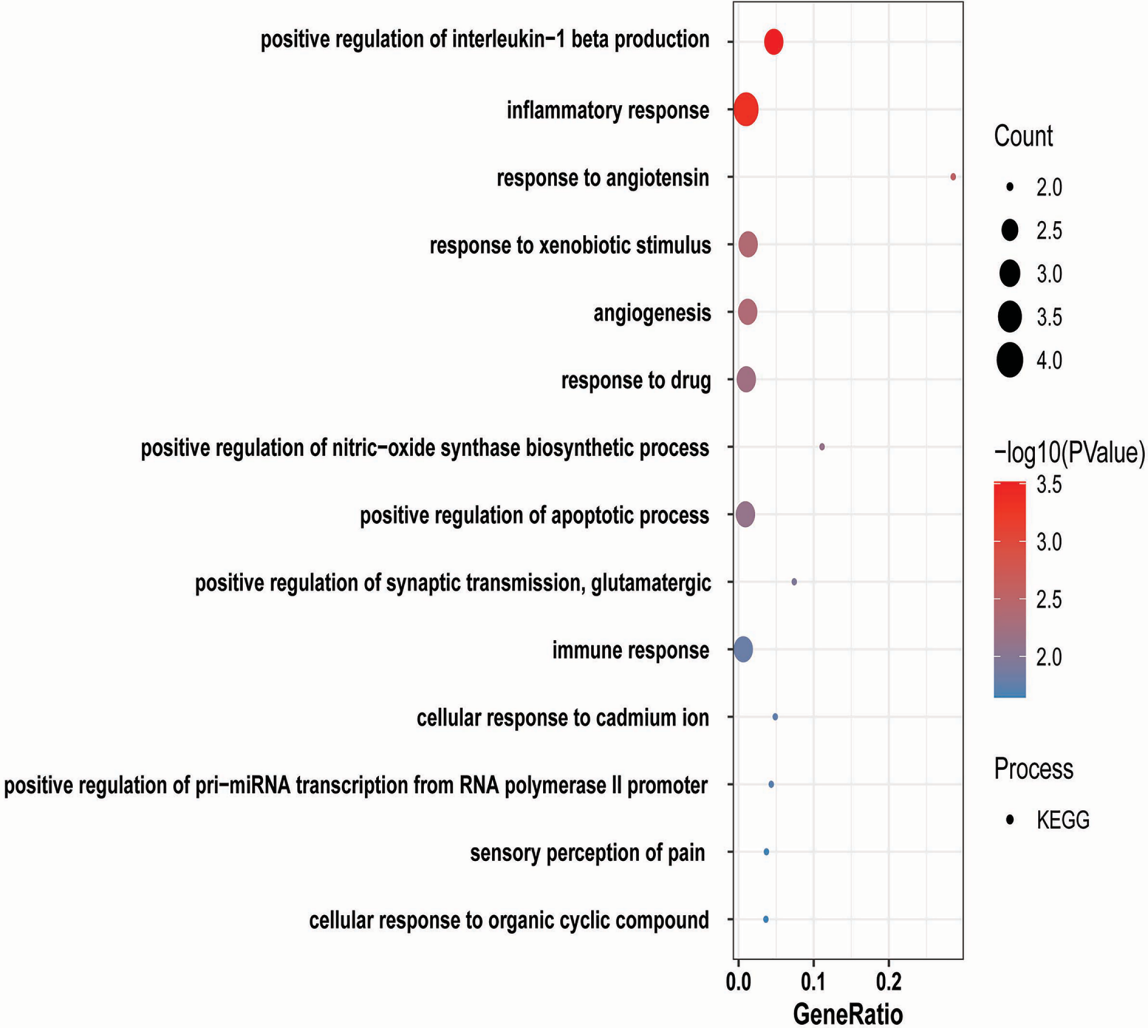
KEGG pathways analysis for KHGs The color of the dots represents the −log_10_(*P-*value), the size of the dots represents the gene count, and the *x*-axis represents the gene ratio.

These KHGs were enriched in pathways related to hsa05140:Leishmaniasis, hsa04933:AGE-RAGE signaling pathway in diabetic complications, hsa05171:COVID-19, hsa04657:IL-17 signaling pathway, hsa04668:TNF signaling pathway, hsa04621:NOD-like receptor signaling pathway, hsa04613:Neutrophil extracellular trap formation, hsa05417:Lipid and atherosclerosis, hsa05022:Pathways of neurodegeneration – multiple diseases, hsa04912:GnRH signaling pathway, has05323:Rheumatoid arthritis, hsa05142:Chagas disease, hsa04625:C-type lectin receptor signaling pathway, hsa04620:Toll-like receptor signaling pathway (Figure 8). The gene enrichment analysis found that most genes are involved in critical biological processes such as apoptotic process, innate immunity response to inflammatory, cell proliferation, etc. Further research on these inferred genes could lead to insights into Amoebiasis infection and prevention processes.

#### Screening for transcription factors

We have identified two TFs, viz JUN and EGR1, among the nine KHGs (JUN, PTGS2, FCGR3A, MNDA, CYBB, EGR1, CCL2, TLR8, and LRRK2). Genes JUN and EGR1 have 150; 88 target genes and 15; 13 regulators, respectively. Genes PTGS2, CYBB, CCL2, and MNDA, have 43, 9, 19, and 2 regulators, respectively (Table 4).

**Table 4:** Transcriptional regulatory relationships unraveled by sentence-based text mining-targets and regulators

S.No.	Gene name	Target genes	Regulators (TFs that regulate)
1	JUN	ABCB1, ACTA1, ALOX12, APOC3, APOM, APP, AR, ATF3, BATF3, BCL2L1, BECN1, BEX2, BRCA, CA2, CCK, CCL2, CCL5, CCND1, CD82, CDK5R1, CDKN1A, CREM, CSF1, CSF2, CSTA, CTGF, CTSL, CXCL8, CYP11A1, CYP19A1, CYP1A2, CYP2J2, DCN, DDIT3, DDX21, EDN1, EGFR, ELN, ETS1, ETS2, EZH2, EZR, F3, FA, FASLG, FGF7, VEGFD, FOSL1, GCLC, GJA1, GSTP1, HEY1, IBSP, IFNB1, IFNG, IL12A, IL12B, IL1A, IL1B, IL2, IL23A, IL24, IL3, IL5RA, IL6, ITGAX, ITGB8, KRT16, LBP, LDHA, LGALS3, LOR, MAP3K1, MAPK8, MAT2A, MEF2D, MELTF, MGMT, MGP, MMP1, MMP12, MMP13, MMP2, MMP20, MMP3, MMP7, MMP9, MSR1, MYB, MYC, NAMPT, NEFL, NEIL1, NFKB2, NGF, NOS2, NOS3, NOX5, NPY, NQO1, NTS, OPRM1, OXTR, PCK2, PDHA1, PDK1, PENK, PGR, PLAT, PLAU, PLAUR, POLD2, PPARA, PPARD, PTGS2, PTN, RARA, RARB, RARG, RELB, REN, RHOB, RUNX2, SLC19A1, SMAD7, SMN1, SOD2, SOX7, SPI1, SPRR1B, SPRR3, STAR, STMN1, SYK, TERT, TFF1, TIMP2, TNC, TNF, TNFRSF10A, TNFRSF1B, TP53, TP73, TXN, UGT2B15, VCAM1, VDR, VEGFA	ABL1, AR, ARNT, ATF2, CREB1, CTNNB1, ESR1, ESR2, GLI1, GLI2, HDAC3, HDAC4, HSF1, MEF2A, MEF2C, MEF2D, NFIC, NFRKB, PARP1, PITX1, RUNX1, SMAD3, SMAD4, TCF4, TNFAIP3, WT1, ZNF382, ZNF383
2	PTGS2	PTGS2 is not a TF.	APC, AR, ATF2, ATF4, CDX1, CDX2, CEBPB, CEBPD, CREB1, CREBBP, CTNNB1, DR1, EGR1, EGR2, ELF3ENO1, EP300, ETV4, FOS, HDAC1, HDAC4, HMGA1, ING4, JUN, JUNB, JUND, NFIL3, NFKB1, NR0B2, PGR, PPARA, PPARG, PPARG, RELA, SETBP1, SP1, STAT1, STAT2, STAT3, STAT6, TCF7L2, TFAP2A, USF1, USF2
3	MNDA	MNDA is not a TF.	SP1, SPI1
4	FCGR3A	FCGR3A is not a TF.	Not Available
5	CYBB	CYBB is not a TF.	CUX1, ELF1, EP300, GATA1, GATA2, IRF1, IRF8, SATB1, SPI1
6	EGR1	ABCB1, ACE, ACHE, AKR1B1, ALOX5, AR, ATF3, BCAR1, CACNA1H, CCND1, CCND2, CD28, CDK5R1, CDKN1A, CHGA, CXCL8, CYP2B6, DMRT1, EAPP, EGFR, EP300, FAP, FAS, FASLG, FCER2, FGF2, FLT1, FN1, GDF15, GDNF, GGPS1, HIF1A, HPSE, HSD11B2, HYAL1, IFNG, IGF2, IL2, IL2RB, IL3, IL6, IMPDH2, KLK3, LDLR, LHB, LMTK2, LTB, MMP14, MYB, NAB2, NFKB1, PCSK2, PDGFB, PDGFC, PLAU, PLAUR, POR, PPARG, PSEN2, PTEN, PTEN, PTGES, PTGS2, PTP4A1, RBL2, SLC4A2, SLC9A3, SOD1, SOX18, SPRY1, STIM1, STMN1, SYN1, SYN2, TBXA2R, TCF4, TFPI2, TGFBR2, TH, TMPO, TNF, TNFSF10, TOE1, TP53, TP73, UBE2S, VEGFA, WNT4	ATF5, BRCA1, ETS1, ETS2, FOXO1, HDAC1, MAML1, NFKB1, NKX2-3, RBMX, RELA, SP1, TP53
7	CCL2	CCL2 is not a TF.	APEX1, ATF4, CEBPA, HDAC2, IRF3, JUN, NFAT5, NFIC, NFKB1, NR1I2, PREB, REL, RELA, SP1, SPI1, STAT1, STAT2, STAT3, XBP1
8	TLR8	Not available	Not available
9	LRRK2	Not available	Not available

### Drugs for key hub genes

We identified approved drugs from DGIdb (https://www.dgidb.org/) [44]. DGIdb is a web-based database of drug–gene interactions and druggable genes. We get drugs against five KHGs (JUN, PTGS2, FCGR3A, CCL2, and LRRK2) as listed in Table 5.

**Table 5:** Drugs for identified KHGs

S.No.	KHGs	Drugs
**1**	**JUN**	SERTRALINE, MECHLORETHAMINE HYDROCHLORIDE, TROPISETRON, BUPROPION HYDROCHLORIDE, AZELASTINE HYDROCHLORIDE, CUPRIC CHLORIDE, CIPROFIBRATE, FENOFIBRATE, VINBLASTINE SULFATE, ATOMOXETINE HYDROCHLORIDE, CINNARIZINE, COLCHICINE, DIPHENHYDRAMINE HYDROCHLORIDE, QUINAPRIL HYDROCHLORIDE, CLOFIBRATE, TRIFLUPROMAZINE HYDROCHLORIDE, GEMFIBROZIL, CLOTRIMAZOLE, VINORELBINE TARTRATE, METHIMAZOLE
**2**	**PTGS2**	IBUPROFEN, FLURBIPROFEN, BALSALAZIDE DISODIUM, KETOROLAC TROMETHAMINE, HYDROXYCHLOROQUINE, FENOPROFEN CALCIUM, AMINOSALICYLATE POTASSIUM, PIROXICAM, BISMUTH SUBSALICYLATE, CYCLOSPORINE, DICLOFENAC, NEPAFENAC, IBUPROFEN LYSINE, NAPROXEN SODIUM, DICLOFENAC POTASSIUM, ACETAMINOPHEN, DICLOFENAC SODIUM, INDOMETHACIN, DICLOFENAC EPOLAMINE, PARECOXIB, KETOROLAC, NIMESULIDE, DIFLUNISAL, BALSALAZIDE, THALIDOMIDE, OXALIPLATIN, OXAPROZIN POTASSIUM, MESALAMINE, SALSALATE, KETOPROFEN, NABUMETONE, FENBUFEN, CARPROFEN, SULINDAC, NAPROXEN, FENOPROFEN, MEFENAMIC ACID, AMINOSALICYLATE SODIUM, SULFASALAZINE, ETORICOXIB, TOLMETIN, OLSALAZINE SODIUM, DEXIBUPROFEN, RALOXIFENE, TOLMETIN SODIUM, MELOXICAM, TENOXICAM, ETODOLAC, ASPIRIN, CAPECITABINE, ATENOLOL, OXAPROZIN
**3**	**FCGR3A**	PENICILLIN G POTASSIUM, ADALIMUMAB, SODIUM CHLORIDE, CETUXIMAB, PREDNISOLONE, FENTANYL, CYCLOSPORINE, INDOMETHACIN, TOCILIZUMAB, THALIDOMIDE, EPOETIN ALFA, DOXORUBICIN, LACTULOSE, TRASTUZUMAB, CIMETIDINE, INFLIXIMAB, ETANERCEPT, RITUXIMAB
**4**	**CCL2**	RISPERIDONE
**5**	**LRRK2**	VANDETANIB, PALBOCICLIB

### Amoebiasis infection revealed miRNAs that target DEG key hub genes

**Figure 9:**
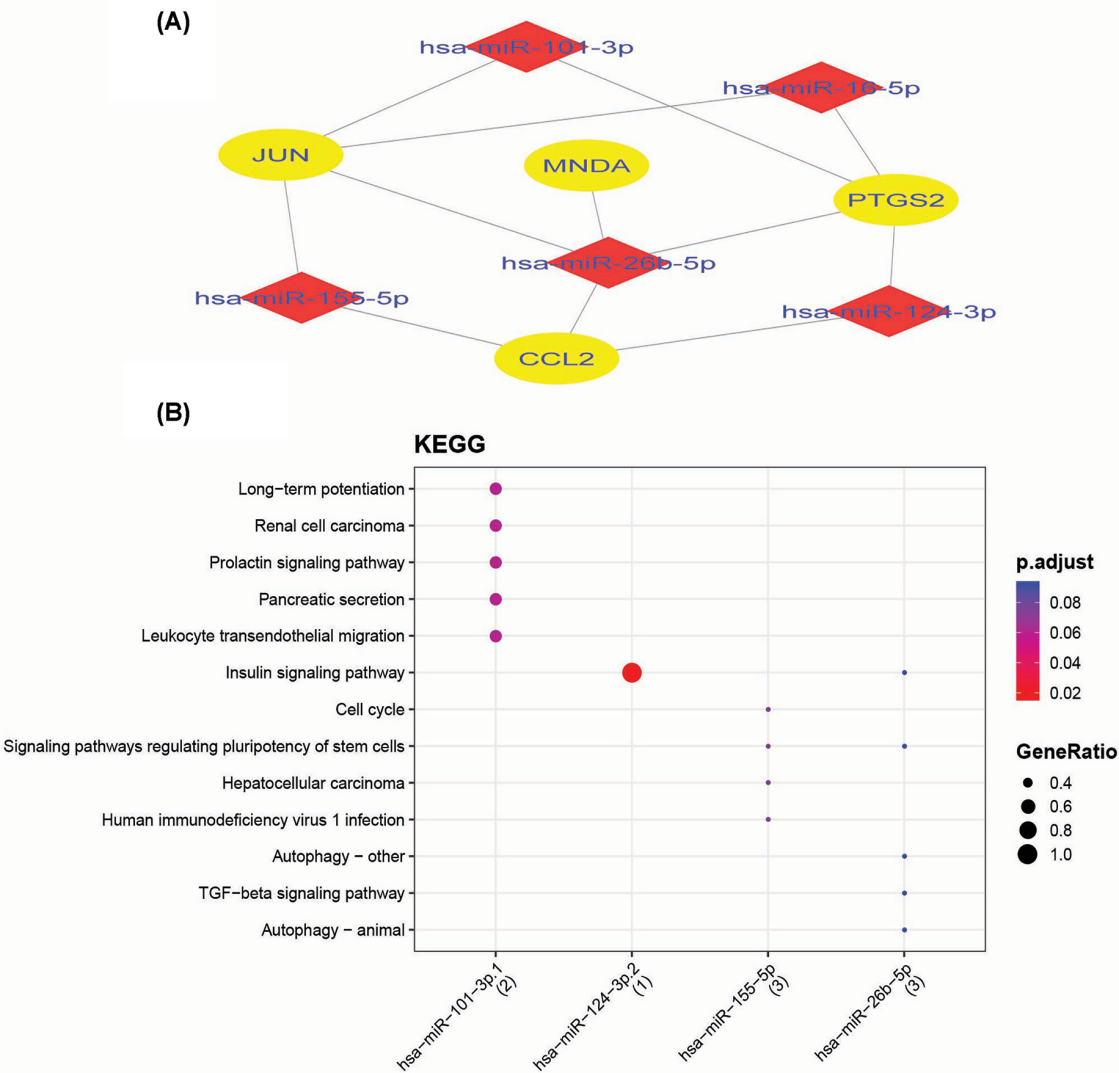
Visualization of miRNAs-gene regulatory network and KEGG pathway analysis (**A**) A miRNA–gene regulatory network contains the KHGs (JUN, PTGS2, CCL2, and MNDA). Green ellipse shape and yellow diamonds represent genes and miRNAs, respectively. (**B**) The dot plot represents the KEGG pathway analysis of miRNAs. The *x*-axis represents the miRNAs, and the *y*-axis represents the KEGG pathways. The color of the dots represents the adjusted *P*-value (FDR), and the size of the dots represents the gene ratio.

**Figure 10:**
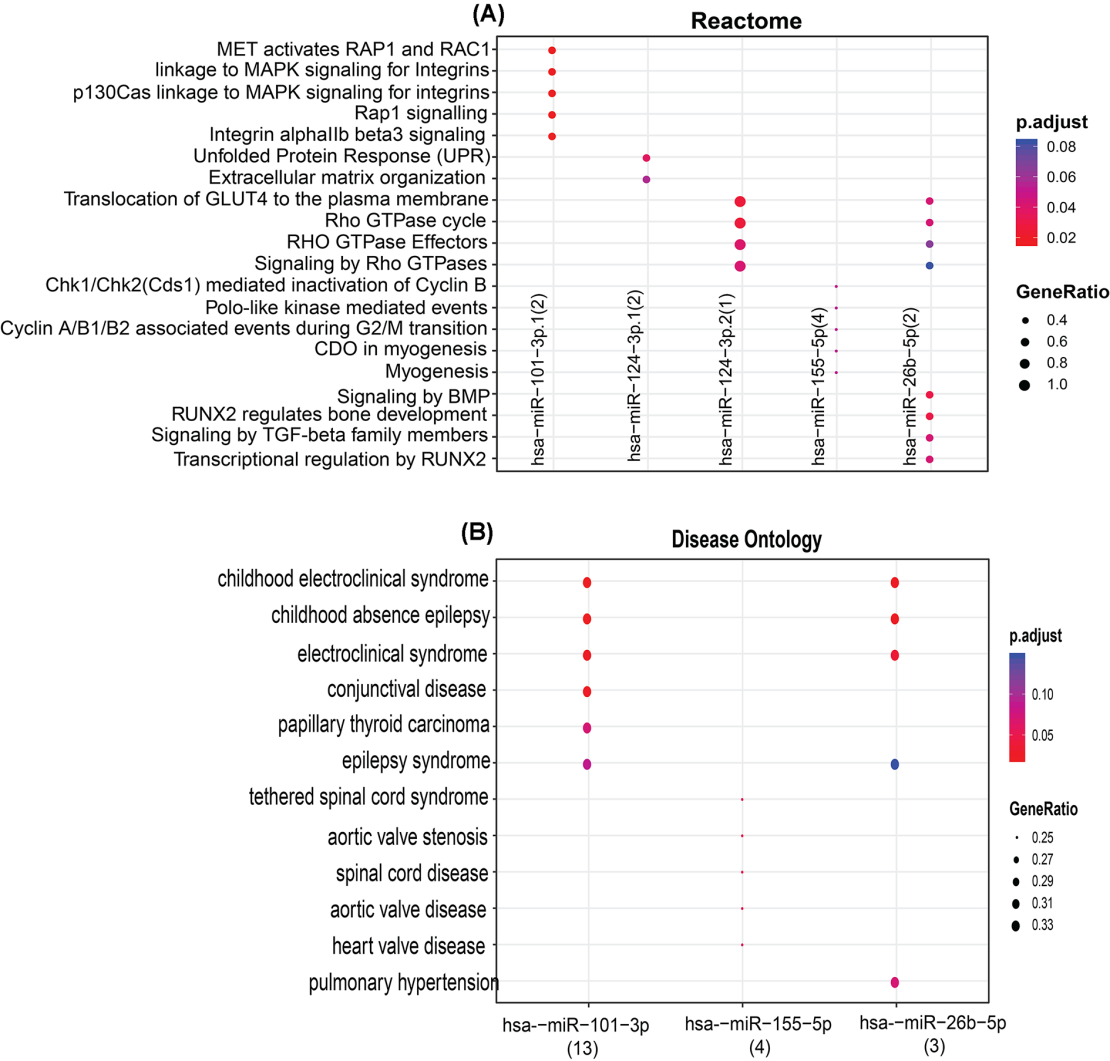
MIENTURNET network analysis of hub genes and differentially expressed miRNAs Dot plot of functional enrichments analysis of target miRNAs resulting from enrichment analysis. The *x*-axis represents the miRNAs, and the *y*-axis represents reactome and disease ontology analysis.

With the help of MIENTURNET [45], we could isolate the targets of KHG–miRNA. JUN, PTGS2, CCL2, and MNDA are the four KHGs having the highest interactions with miRNAs. The networks of interactions between KHGs and miRNAs were built (Figure 9A). hsa-miR-155-5p, hsa-miR-101-3p, hsa-miR-124-3p, hsa-miR-26b-5p, and hsa-miR-16-5p are among the important miRNAs shown to be targeted by KHGs. In Amoebiasis infection, the enrichment of the five differentially expressed miRNAs detected using KHGs were substantially linked with differently expressed miRNAs (*P* = 0.05 and FDR = 0.1). The differentially expressed miRNAs’ possible function was discovered using disease ontology, WikiPathways, Reactome, and KEGG pathway analysis. These miRNAs were shown to be grouped in a variety of pathways, with a focus on long-term potentiation, renal cell carcinoma, prolactin signaling pathway, pancreatic secretion, leukocyte transendothelial migration, neurotrophin signaling pathway, platelet activation, Cushing syndrome, JAK-STAT signaling pathway, chemokine signaling pathway, focal adhesion, Rap1 signaling pathway, lipid and atherosclerosis, cAMP signaling pathway, Ras signaling pathway, MAPK signaling pathway, insulin signaling pathway, cell cycle, signaling pathways regulating pluripotency of stem cells, hepatocellular carcinoma, human immunodeficiency virus 1 infection, TGF-β signaling pathway, insulin signaling pathway, autophagy – animal, signaling pathways regulating pluripotency of stem cells, Spinocerebellar ataxia, mTOR signaling pathway, Hippo signaling pathway, transcriptional misregulation in cancer, Huntington disease, amyotrophic lateral sclerosis, Alzheimer’s disease, and pathways of neurodegeneration – multiple diseases (Figures 9B and 10). WikiPathways analysis discovered possible function as endoderm differentiation, mesodermal commitment pathway, bone morphogenic protein (BMP) signalling and regulation, development of pulmonary dendritic cells and macrophage subsets, BMP2-WNT4-FOXO1 pathway in human primary endometrial stromal cell differentiation, interactome of polycomb repressive complex 2 (PRC2), regulation of Wnt/B-catenin signaling by small molecule compounds, BMP signaling pathway in eyelid development, signaling of hepatocyte growth factor receptor, angiogenesis, nanoparticle triggered autophagic cell death, differentiation of white and brown adipocyte, common pathways underlying drug addiction, IL-4 signaling pathway, and target of rapamycin (TOR) signaling. Reactome pathways analysis implicated MET activates RAP1 and RAC1, GRB2:SOS provides linkage to MAPK signaling for integrins, p130Cas linkage to MAPK signaling for integrins, Rap1 signaling, Chk1/Chk2(Cds1) mediated inactivation of Cyclin B: Cdk1 co…, Integrin αIIb β3 signaling, integrin signaling, signaling by BMP, Polo-like kinase mediated events, RUNX2 regulates bone development, signaling by high-kinase activity BRAF mutants, platelet aggregation (plug formation), MAP2K and MAPK activation, signaling by moderate kinase activity BRAF mutants, and paradoxical activation of RAF signaling by kinase. Disease childhood electroclinical syndrome, childhood absence epilepsy, tethered spinal cord syndrome, conjunctival disease, aortic valve stenosis, spinal cord disease, heart valve disease, pulmonary hypertension, congenital heart disease, papillary thyroid carcinoma, intellectual disability, thyroid carcinoma, epilepsy syndrome, thyroid cancer, specific developmental disorder, liver cirrhosis, pancreatic cancer, autonomic nervous system neoplasm, neuroblastoma, peripheral nervous system neoplasm, adenoma, brain disease, developmental disorder of mental health, non-small cell lung carcinoma, cell-type benign neoplasm, and others were discovered by miRNA ontology study (Figure 10).

## Discussion

Investigating transcriptional changes in *E. histolytica* infected cells (Amoebiasis) yields fresh insights into the infection’s host response. Despite significant advancements, the specific molecular pathways of Amoebiasis and its development remain unknown. As a result, it’s critical to research the mechanism to pinpoint molecular targets for diagnosis and treatment. The search for DEGs has intensified in recent decades, and differential expression of DEGs has become extensively distributed.

This work extracted raw gene expression data from four GSE series from the GEO dataset, yielding 451 specific DEGs, including 193 up-regulated and 258 down-regulated genes that met the *P*-value and folded change cut-off criteria. The KEGG pathways results show that the down-regulated DEGs mainly were linked with Pertussis, IL-17 signaling pathway, chemokine signaling pathway, drug metabolism-cytochrome P450, drug metabolism-cytochrome P450, Human cytomegalovirus infection, viral protein interaction with cytokine and cytokine receptor, chemical carcinogenesis-DNA adducts, Leishmaniasis, complement and coagulation cascades, Herpes simplex virus 1 infection, and steroid hormone biosynthesis. In comparison, the up-regulated DEGs were enriched in human T-cell leukemia virus 1 infection, epithelial cell signaling in *H. pylori* infection, retinol metabolism, C-type lectin receptor estrogen signaling pathway, TNF signaling pathway, porphyrin metabolism, biosynthesis of cofactors, GnRH signaling pathway, and cytokine–cytokine receptor interaction. These findings are also helpful in studying molecular relationships in Amoebiasis.

Furthermore, the Amoebiasis network was constructed from up- and down-regulated genes (DEGs).

JUN, PTGS2, MNDA, FCGR3A, CYBB, EGR1, CCL2, TLR8, and LRRK2 were the most influential KHGs of the *E. histolytica* infection in our study. The differential expression of four biomarkers (JUN, PTGS2, CCL2, and MNDA), which are known to be direct targets of the *E. histolytica* genome, was verified by protein interaction network analysis of *E. histolytica* infected DEGs. The most likely disease target genes, these critical KHGs, serve as the backbone of network activities and regulators.

The JUN gene was up-regulated when *E. histolytica* infection patients were compared with healthy controls. The gene JUN is a TF that recognizes and binds to the AP-1 consensus motif 5′-TGA [GC]TCA-3′ [46]. JUN forms an AP-1 transcription complex by heterodimerizing with FOS family proteins, boosting its DNA binding activity to the AP-1 consensus sequence 5′-TGA [GC]TCA-3′ and transcriptional activity. Binding to the AP-1 promoter site of FASLG/CD95L and promoting its transcription in response to activation of the TCR/CD3 signaling pathway, together with FOSB, plays a role in activation-induced cell death of T cells [47]. When HIPK3 phosphorylates JUN, it promotes the activity of NR5A1, resulting in enhanced steroidogenic gene production in response to cAMP signaling pathway stimulation [48]. JUN is involved in the transcriptional activation of USP28 in colorectal cancer (CRC) cells by activated KRAS. In CRC cells, JUN interacts with the promoter of USP28 [49].

PTGS2 gene represents prostaglandin-endoperoxide synthase 2. The primary enzyme in prostaglandin formation, prostaglandin-endoperoxide synthase (PTGS), commonly known as cyclooxygenase, is a dioxygenase and peroxidase. PTGS is divided into two isozymes: constitutive PTGS1 and inducible PTGS2 [50], which regulate the expression and tissue distribution differently [51]. This gene encodes inducible isozyme. It’s triggered by certain stimuli, implying that it’s in charge of prostanoid production, which is implicated in inflammation and mitogenesis [52]. In the production route of prostanoids, a class of C20 oxylipins generated primarily from arachidonate, dual cyclooxygenase, and peroxidase play a vital role in the inflammatory response [53]. During both sterile and viral inflammation, it produces resolution phase interaction products (resolvins) [54]. Fc-binding subunit that forms functional signaling complexes with CD247 and FCER1G adapters. Following antigen–IgG complex contact, phosphorylation of immunoreceptor tyrosine-based activation motif (ITAM)-containing adapters occurs, followed by activation of phosphatidylinositol 3-kinase signaling and persistent intracellular calcium increase, which leads to NK cell activation [55]. In the absence of receptor phosphorylation, ITAM-dependent signaling activates phosphatidylinositol 3-kinase signaling, which leads to cell degranulation [56]. In contrast, in the presence of receptor phosphorylation, it mainly activates phosphatidylinositol 3-kinase signaling, which leads to cell degranulation [57].

Myeloid cell nuclear differentiation antigen (MNDA) gene acts as a transcriptional activator/repressor in the myeloid lineage and plays a role in the granulocyte/monocyte cell-specific response to interferon. It stimulates the DNA binding of the transcriptional repressor protein YY1. MNDA is a protein-coding gene that is found in myeloid cells. Nodal marginal zone lymphoma and Parkinson’s disease 4, autosomal dominant, is two diseases linked to MNDA. NF-κB signaling and the innate immune system are two of its linked pathways.

FCGR3A is the receptor for immunoglobulin γ's invariable Fc segment (IgG). Antibody-dependent cellular cytotoxicity is triggered by the binding of clustered antigen–IgG complexes exhibited on cell surfaces (ADCC). In the absence of an antigenic trigger, it does not bind free monomeric IgG, preventing incorrect effector cell activation [58]. This protein mediates IgG effector actions on natural killer (NK) cells. Binds antigen–IgG complexes formed during infection and activates cytokine production and degranulation in NK cells, limiting viral load and propagation. It is involved in developing memory-like adaptive NK cells capable of producing large amounts of IFNG and removing virus-infected cells efficiently via ADCC [59,60].

The CYBB gene directs the production of a protein called cytochrome b-245, β chain. This protein is a component of a group of proteins that make up the NADPH oxidase enzyme complex, which is vital for the immune system. The α (CYBA) and β (CYBB) chains make up cytochrome b (-245). It’s thought to be a vital component of the phagocyte’s microbicidal oxidase system. One of five biochemical deficiencies linked to chronic granulomatous illness has been identified: CYBB deficiency (CGD) [61]. The activity of phagocyte NADPH oxidase is reduced in this disease, which allows neutrophils to phagocytize germs but not destroy them in the phagocytic vacuoles. The failure to transfer activated oxygen into the phagocytic vacuole results from the cell’s incapacity to boost its respiration [62].

The CCL2 gene (C-C motif chemokine ligand 2) codes for a protein. Neural tube defects and human immunodeficiency virus type 1 are two diseases linked to CCL2. Folate metabolism and the TGF-β pathway are connected processes [63,64]. Protein kinase activity and heparin-binding are examples of GO annotations for this gene. It binds to CCR2 and activates it, resulting in a robust chemotactic response and calcium ion mobilization within the cell [65]. It may be engaged in the recruitment of monocytes into the artery wall during the atherosclerosis disease process [66]. On the q-arm of chromosome 17, this gene is one of the numerous cytokine genes. Chemokines are a kind of released protein that has a role in immune regulation and inflammation. Based on the arrangement of N-terminal cysteine residues in the mature peptide, the superfamily is classified into four subfamilies. This chemokine belongs to the CC subfamily, defined by two cysteine residues located next to each other. Monocytes and basophils respond to this cytokine, whereas neutrophils and eosinophils do not. Psoriasis, rheumatoid arthritis, and atherosclerosis have been linked to monocytic infiltrates. It attaches to the CCR2 and CCR4 chemokine receptors.

TLR8 gene produces a Toll-like receptor (TLR) protein, which is involved in pathogen identification and innate immune activation. TLRs exhibit structural and functional commonalities from drosophila to humans. They identify pathogen-associated molecular patterns on infectious pathogens and mediate the generation of cytokines needed for successful immunity development. The expression patterns of the various TLRs are distinct. This gene is found on chromosome X near another family member, TLR7, and is primarily expressed in lung and peripheral blood leukocytes. TLR8 (Toll-like receptor-8) is a gene that codes for a protein. TLR8 is linked to immunodeficiency 98 and other diseases. Anemia, autoimmune hemolytic, autoinflammation, X-linked TRAF6-mediated NF-kB, and MAP kinase up-regulation in response to TLR7/8 or 9 activations is related mechanisms. Controls the host’s immunological response against diseases by recognizing RNA degradation products unique to bacteria, which are processed by RNASET2 [67]. When it binds to agonists, it dimerizes, bringing the TIR domains of the two molecules into close contact, resulting in the homotypic recruitment of TIR-containing downstream adapter MYD88 [68]. The Myddosome signaling complex, which includes IRAK4, IRAK1, TRAF6, and TRAF3, then activates downstream TFs NF-kB and IRF7, producing proinflammatory cytokines and interferons, respectively [69].

The LRRK2 gene encodes a protein featuring an ankyrin repeat region, a leucine-rich repeat (LRR) domain, a kinase domain, a DFG-like motif, RAS, and GTPase, an MLK-like domain, and a WD40 domain. It belongs to the LRR kinase family. The protein is primarily found in the cytoplasm, although it can also be found in the mitochondrial outer membrane. Parkinson’s disease has been linked to mutations in this gene-8. ncRNAs engaged in Wnt signaling in hepatocellular carcinoma and the MAPK signaling pathway are two of the associated pathways [70]. Protein homodimerization and transferase activity, which transfer phosphorus-containing groups, are two GO annotations associated with this gene.

EGR1 codes for a zinc-finger protein that belongs to the EGR family of C2H2-type zinc-finger proteins. It’s a transcriptional regulator that’s found in the nucleus. Differentiation and mitogenesis are dependent on the products of the target genes it activates [71]. Studies identified this gene as a cancer suppressor gene [72,73]. These genes are also involved in several other life-threatening diseases, including various types of cancer, Alzheimer’s, etc.

MiRNAs regulate the expression of host genetic components required by parasites. miRNAs appear to have a post-transcriptional influence on gene expression, affecting various biological activities, including disease development and progression. miRNAs are also involved in regulating parasite-host interactions [74,75]. This investigation discovered that various host miRNAs target hsa-miR-155-5p *E. histolytica*-infected KHGs. hsa-miR-155-5p, hsa-miR-101-3p, hsa-miR-124-3p, hsa-miR-26b-5, and hsa-miR-16-5p are among the important miRNAs shown to be targeted by (JUN, PTGS2, CCL2, and MNDA) KHGs. As a result, differential expression and network analyses were used to examine the gene expression profile after *E. histolytica* infection. miRNAs hsa-miR-155-5p, hsa -miR-101-3p, hsa-miR-124-3p, hsa-miR-26b-5p, and hsa-miR-16-5p present us a narrow window of novel therapeutic opportunity to target genes in *E. histolytica* infection. KEGG pathway enrichment depicted that miRNAs hsa-miR-155-5p, hsa-miR-101-3p, and hsa-miR-26b-5p are clustered in long-term potentiation, renal cell carcinoma, prolactin signaling pathway, pancreatic secretion, leukocyte transendothelial migration, insulin signaling pathway TGF-β signaling pathway, human immunodeficiency virus 1 infection, Signaling pathways regulating pluripotency of stem cells, etc. (Figure 9B). Reactome enrichment depicted that miRNAs-miR-101-3p1, hsa-miR-124-3p.1, hsa-miR-124-3p.2, hsa-miR-155-5p, and hsa-miR-26b-5p are clustered in transcriptional regulation by RUNX2, signaling by TGF-β family members, RUNX2 regulates bone development, signaling by BMP, Rho GTPase cycle, signaling by Rho GTPases, RHO GTPase effectors, Rho GTPase cycle, MET activates RAP1 and RAC1, etc. (Figure 10). MiRNAs hsa-miR-101-3p.1, hsa-miR-155-5p, and hsa-miR-26b-5p have also been grouped in pulmonary hypertension, heart valve disease, aortic valve disease, spinal cord disease, aortic valve stenosis, tethered spinal cord syndrome, epilepsy syndrome, papillary thyroid carcinoma, conjunctival disease, electroclinical syndrome, and other disorders, according to miRNA ontology studies (Figure 10).

The transcriptional and post-transcriptional regulation of gene function is equally important. As a result, we investigated the miRNA-KHGs and TF-KHGs networks to understand better the regulatory behavior of the KHGs found. Gene transcription is regulated by TFs, which can be coordinated through genes with related activities. miRNA, on the other hand, are potent post-transcriptional regulators of transcript levels; however, it should be noted that other less potent and less well-defined groups of non-coding RNAs also affect transcript levels post-transcriptionally. Thus, we employed miRNA and TF targets among the KHGs involved in Amoebiasis to determine their targets. This research found some TFs with the most substantial ties to KHGs.

Our findings revealed that these TFs formed a linked regulatory network with KHGs, implying that dynamic changes in these TFs’ activities appear in Amoebiasis and may play a key role in regulating the gene function and expression of KHGs linked to the appearance and progression of Amoebiasis.

According to this study, as a result, the discovered few KHGs may operate as therapeutic targets for Amoebiasis in the future. There are some drawbacks, such as the small sample size. Furthermore, due to a lack of experimental investigations and validations, we may be unable to dig deeper into how KHG–miRNA networks affect Amoebiasis diagnosis and therapy. Despite these constraints, this analysis may deliver more accurate results based on the integrated bioinformatics analysis than single dataset studies.

## Conclusions

We used an integrated analysis based on four microarray gene expression profiles of Amoebiasis and healthy controls to identify DEGs and their biological functions and a pathways enrichment analysis. The topological features of the gene interaction network were studied, and significant Amoebiasis KHGs were discovered. We also built a network of miRNA–KHGs and TF–KHGs. The significance of KHGs was revealed in our study. Next, we identified approved drugs against these KHGs. As a result, given the preventative effect of immune response on the emergence of Amoebiasis, these genes are expected to play a substantial role.

## Limitation of study

However, the sample size is limited, and more research is needed to confirm the expression and function of the KHGs found in Amoebiasis. Furthermore, lncRNA analysis is missing in this analysis. LncRNAs have been implicated in various functions, including controlling intestinal epithelial cell death, lipid metabolism, and cell–cell interactions, increasing inflammation, and regulating T-cell function. LncRNAs are linked to the normal development and pathogenesis of a variety of disorders [76–78]. As a result, lncRNAs play a crucial role in organisms’ growth, development, aging, and death [79]. Therefore, further study is needed using other mathematical techniques, e.g., Petri Nets, and Graph Theory [80–85], to explore the critical role of lncRNAs in intestinal disorder and their potential roles as a therapy.

## Statistical packages and software tools used in this study

R version 4.1.2 (2021-11-01) was used.

LIMMA package was used to analyze data and quantify DEGs.

Cytoscape 3.8.2 was used for network data visualization.

Cytoscape plugin CytoNCA was used for centrality measurement.

Cytoscape plugin CytoHubba was used for MCC, DMNC, MNC, and EPC properties.

Server (http://biit.cs.ut.ee/gprofiler/convert) was used for probe ID conversion.

TRRUST (v2.0) server (https://www.grnpedia.org/trrust/) was used for elucidating the TF–target interaction.

DAVID 6.8 Web server (https://david.ncifcrf.gov/) used for GO-enrichment.

STRING v11 database (https://string-db.org/) was used to construct a PPI network.

MIENTURNET(http://userver.bio.uniroma1.it/apps/mienturnet/) server was used to isolate the targets of KHG–miRNA.

DGIdb (https://www.dgidb.org/) web-based database of drug–gene interactions was used for drug identification.

## Supplementary Material

Supplementary Files S1-S4

## Data Availability

All data are available in the NCBI GEO database; https://www.ncbi.nlm.nih.gov/geo/

## Abbreviations

BMP: bone morphogenic protein
DEG: differentially expressed gene
ITAM: immunoreceptor tyrosine-based activation motif
KHG: key hub gene
LRR: leucine-rich repeat
NK: natural killer
PPI: protein–protein interaction
PRC2PR: polycomb repressive complex 2
TF: transcriptional factor
TLR: Toll-like receptor
